# The Fight against Infectious Diseases: The Essential Role of Higher-Order Thinking and Problem-Solving

**DOI:** 10.3390/jintelligence10010014

**Published:** 2022-02-21

**Authors:** Juliana Gottschling, Florian Krieger, Samuel Greiff

**Affiliations:** 1 Department of Behavioral and Cognitive Science, University of Luxembourg, Maison des Sciences Humaines, 11, Porte des Sciences, L-4366 Esch-sur-Alzette, Luxembourg; juliana.gottschling@uni.lu; 2 Methods of Educational Research, Faculty of Rehabilitation Sciences, TU Dortmund University, Emil-Figge-Str. 50, 44227 Dortmund, Germany; florian.krieger@tu-dortmund.de

**Keywords:** problem-solving, higher-order thinking, real-world environments, infectious diseases

## Abstract

The development of a vaccine marks a breakthrough in the fight against infectious diseases. However, to eradicate highly infectious diseases globally, the immunization of large parts of the population is needed. Otherwise, diseases, such as polio, measles, or more recently COVID-19, will repeatedly flare-up, with devastating effects on individuals and, in the worst case, on significant shares of the world population. For example, polio has been almost eradicated over the past three decades through an unprecedented global effort, but complete immunization has not yet been achieved. In this article, we use polio as an example to show how the global effort of developing and administering a vaccine can be understood as solving a complex problem since it involves cultural, political, and geographical barriers that demand solutions in dynamically changing and highly versatile environments. Referring to the literature on problem-solving, higher-order thinking, and complex reasoning, we demonstrate how the ability to deal with real-world environments that are complex and dynamically changing, adapting initial solutions to new circumstances and collaborate efficiently with others, has been essential for this endeavor. We argue that problem-solving abilities form one basis for solving consequential world problems.

## 1. Introduction

This Special Issue addresses the question of “How Intelligence Can Be a Solution to Consequential World Problems”. A multitude of consequential world problems are facing humanity today—problems that are not just important but affect all people living on our planet. Climate change and the COVID-19 pandemic are just two examples that quickly come to mind. What consequential world problems have in common is that there exists no single, simple solution to solve them. Consequently, problem-solving skills are required to solve them, which are often subsumed under the umbrella term *intelligence*. Broad definitions of intelligence include adapting to the environment in novel situations to overcome obstacles (e.g., Neisser et al. 1996) attributing to intelligence an integral role in tackling consequential world problems. In fact, intelligence research has a long history, and intelligence is seen as one of the most important ingredients for life success (e.g., Gottfredson 2002; Roth et al. 2015). However, there are also concerns that essential skills for solving problems in our current society are not fully reflected in narrow definitions of intelligence and, thus, are not part of the standardized intelligence tests (Halpern and Dunn 2021; Stanovich 2009; and Stanovich 2014). For instance, although problem-solving skills and intelligence are both theoretically and empirically related (e.g., Greiff et al. 2014 and Stadler et al. 2015), there are certain problem-solving skills, which are essential for solving consequential world problems but not reflected in traditional intelligence tests (for instance, detecting and controlling autonomous changes in problems; see Stadler et al. 2019). In the current article, we review the role of higher-order thinking (HOT) skills as one promising candidate reflecting integral, established problem-solving skills. As one example from the set of consequential world problems, we focus on the approach to eradicate the poliovirus. The main message of this article is not that intelligence is irrelevant for solving consequential world problems, but that for solving these problems integral problem-solving skills, such as HOT, need to be considered on a more fine-grained level, which might inspire both (1) policymakers to continue the efforts for implementing and adapting these skills in school curricula, and (2) researchers to further consider these skills in narrow descriptions of intelligence and its assessments.

## 2. Higher-Order Thinking and Problem-Solving

Higher-order thinking refers to the skills that go beyond memorizing and recalling information, but instead emphasize the reasoned application of knowledge in a diverse set of contexts (Brookhart 2010, and Greiff and Martin 2015). Individuals with strong HOT skills can analyze and evaluate complex and possibly new information; categorize, manipulate and combine facts; search for (innovative) solutions; understand concepts; transfer and connect knowledge; draw upon a broad perspective to solve problems; and generate ideas and develop insightful reasoning (Anderson et al. 2001). HOT skills are often described in educational contexts as belonging to the three highest levels in Bloom’s taxonomy, i.e., analyzing, evaluating, and creating, and are seen as indispensable for 21st-century learning.

HOT typically becomes relevant in unfamiliar situations, when we are confronted with a given state that we want to transform into a goal state, but the steps and actions required to do so are unknown (Greiff and Martin 2015). A situation in which our previous experiences and routine solutions are not applicable or not sufficient to reach a goal state is defined in cognitive psychology as a *problem*. Thus, to reach the desired goal state, we need to engage in the process of problem-solving. Problem-solving refers to any kind of goal-directed sequence of cognitive operations (Anderson 1982) by which an individual moves from an initial situation, in which the problem is identified and represented, to a desired goal state by manipulating the components of the problem to reach a conclusion or to solve the problem (Jonassen 2000; Osman 2017; and VandenBos 2007). On a generic level, problem-solving includes the process of constructing a mental model (i.e., a mental representation) of the problem, the search for solutions, and the implementation and monitoring of the solutions (Gick 1986). Thus, any problem-solving process requires a certain amount of complex thinking, and therefore involves HOT, making the two closely related (Greiff and Martin 2015).

Since problem-solving is a situationally specific activity, the demands of the problem situation are not uniform and often require non-routine solutions. In other words, problems are not equivalent in content, form, or process (Jonassen 2000) and thus require a set of problem-solving skills all involving HOT, each with a specific focus, to reach a solution. We argue below those three problem-solving skills, namely complex, adaptive, and collaborative problem-solving, are key competencies when dealing with consequential world problems. Before making this argument, we must first take a closer look at the characteristics of consequential world problems.

## 3. Consequential World Problems and How They Relate to Problem-Solving Skills

The United Nations lists no fewer than 22 issues that it considers to be most urgent at present, e.g., aging, atomic energy, climate change, poverty eradication, health, migration, or water (United Nations 2021). Common to these problems is that they tend to be ill-structured (i.e., with no clear path to a solution, no single correct solution, and various possible points for intervention), dynamically changing, ambiguous, and intransparent (i.e., not all the information is given from the beginning and data are often missing or invalid). In addition, there are often many parties involved with competing interests and needs, not all of which can be satisfied, and they are often closely tied to other issues^1^ (see the Nautilus Institute for Security and Sustainability 2008; Sternberg et al. 2019). Considering these characteristics, it becomes clear that it indeed takes more than an IQ and declarative knowledge to tackle these types of problems, bringing us back to the question of particularly relevant problem-solving skills.

First, when problems are not static but unfamiliar and dynamically changing, such as the global problems described above, complex problem-solving (CPS; e.g., Greiff et al. 2013) comes into play in finding a solution. CPS describes skills in successfully coping with new and rapidly changing problem situations in which no routine solution is available (Mayer and Wittrock 2006). The main characteristics of complex problems are that not all of the information is given at the beginning (intransparency), that there are multiple input variables affecting the output variables (complexity), and that some output variables change autonomously, without user input (dynamics; Greiff et al. 2013). To solve a complex problem, the problem solver must first acquire knowledge by actively exploring the problem and then apply this acquired knowledge to achieve specific goals (Funke 2001; Wüstenberg et al. 2012).

Closely related but not equivalent to CPS is adaptive problem-solving (APS; Greiff et al. 2017). APS reflects the ability “to react to unforeseen changes and new information in a flexible and adaptive way” (Greiff et al. 2021), which is certainly of great value when it comes to consequential world problems. Since APS involves the ability to achieve one’s goals in a dynamic situation, in which a solution procedure is not immediately available, it directly meets the requirements of the described characteristics of global problems. More concretely, consequential world problems require the problem-solver to consider the various resources in different information environments (be they physical, social, or digital) in addition to their own mental activities, because the former often change dynamically during problem-solving. These changes occur because consequential world problems typically consist of a set of complex, interacting issues that evolve in a dynamic social, political, and cultural context. New problems often emerge as an unintended result of the problem-solver’s actions and attempts to understand the problem. Consequently, the constant monitoring of the problem situation is needed, as well as an adaptation as necessary. Hence, adaptive problem-solvers must engage not only in cognitive, but also strongly in metacognitive processes to define the problem, search for information, and apply a solution (Greiff et al. 2017). Metacognitive processes describe the ability to adjust one’s comprehension of the problem as necessary, evaluate the possible solutions, and monitor the progress toward set goals (Greiff et al. 2021).

This process of adaptation and reflection also requires rational thinking. This means that the problem solver needs to reflect whether the solution can be achieved by efficient means (instrumental rationality) and whether the beliefs of the problem solver correspond to real-world evidence (epistemic rationality; Stanovich 2014 and Stanovich 2016).

In addition, addressing the consequential world problems always involves different groups of people, whether in collaborating on a solution and benefitting from each other’s knowledge or negotiating compromises between groups with different interests. This is where collaborative problem-solving (ColPS; Graesser et al. 2018) skills are of great value. ColPS has been defined in different ways (see Han et al. 2021), but one common definition describes ColPS as “the capacity of an individual to effectively engage in a process whereby two or more agents attempt to solve a problem by sharing the understanding and effort required to come to a solution and pooling their knowledge, skills, and efforts to reach that solution” (Organization for Economic Co-operation and Development; OECD 2017). In a sense, ColPS reflects the application of individual problem-solving skills in a group context (Greiff 2012). ColPS skills are relevant in the contexts in which we interact with others, whether at school, at work, or in our personal lives (Graesser et al. 2018; OECD 2017; and Stadler et al. 2020).

As we have seen, consequential world problems have specific characteristics that require specific skills among problem-solver(s) to be addressed appropriately. We argued that among the many different problem-solving skills, three might be of particular value, namely complex, adaptive, and collaborative problem-solving skills. It is important to emphasize that we do not claim that these are the only relevant skills; however, we do believe that they, together with the associated HOT skills, can guide the process of reaching the desired goal state, i.e., the (partial) solution to a consequential world problem. Moreover, these skills do not function independently, but rather are interlinked and applied in close conjunction with each other. We will now apply what we have derived theoretically to the concrete example of infectious diseases to demonstrate how HOT and problem-solving skills come into play.

## 4. The Global Fight against Polio: An Example of Successful Problem-Solving in Action

Poliomyelitis (polio) is a highly infectious viral disease transmitted mainly via the fecal–oral route. The virus replicates in the intestine, from where it can enter the nervous system and can cause paralysis (World Health Organization; WHO 2021). Polio predominantly affects children under five years of age. Even before the COVID-19 pandemic, infectious diseases, such as polio, were one of the most critical issues facing humanity. They affect the entire world and pose a constant health and economic threat. Thus, infectious diseases are a consequential world problem that exhibits the typical characteristics of being ill-structured, dynamically changing, intransparent and complex, ambiguous, and solvable only through internationally coordinated and interdisciplinary approaches (Becker et al. 2006). An example of such an approach is the Global Polio Eradication Initiative (GPEI).^2^ The GPEI is one of the largest global health initiatives of all time (Cochi et al. 2014) and has set the goal of eradicating polio worldwide. When GPEI was launched in 1988, wild poliovirus had spread to more than 125 countries and was paralyzing about 1000 children per day (Bill & Melinda Gates Foundation 2021). Over the past three decades, the number of cases declined by more than 99%, thanks to extensive vaccination efforts. As of today, Pakistan and Afghanistan are the only countries in which wild polio occurs (Bill & Melinda Gates Foundation 2021). When examining the efforts of eradicating the poliovirus more closely, it becomes evident that there were many obstacles, uncertainties, and overlapping issues along the way (not yet completed), and that HOT and related problem-solving skills were involved in each step of the process.

The starting situation thus met all the main criteria of a complex problem as introduced above. That is, the situation was intransparent and complex, and no uniform approach had yet been established in the countries concerned. In detail, in many countries, there was a lack of information among both the population and governments, and there were multiple, variable, and often uncontrollable factors at play that influenced the achievement of the major goal of eradicating polio worldwide. In fact, the (causal) relationships between multiple interrelated variables, such as the acceptance of the vaccination campaign, religious beliefs, current political situations, or logistics, had to be uncovered and (rational) actions needed to be planned and executed based on these insights. One example of multiple variables being involved is that insecurity in certain regions led to logistic obstacles potentially impacting the vaccine quality (e.g., Ahmad et al. 2020).

Indeed, the initial solution process was akin to solving a complex problem: The GPEI first acquired knowledge about the social, cultural, political, and religious barriers that may exist in a country and that need to be understood to improve vaccination coverage. This knowledge was then applied to find ways to work with local political leaders and health professionals (Bill & Melinda Gates Foundation 2021, and Cochi et al. 2014). The strategy developed to address the problem involved training and mobilizing millions of volunteers and health workers. It was also recognized as critical to reaching households not reached by other health initiatives to completely eradicate polio.

Despite the great initial success with this strategy even in developing countries, polio outbreaks continued to emerge in certain areas, such as parts of Africa or Pakistan. In other words, the situation frequently changed dynamically during the problem-solving process. For instance, the migration of unvaccinated people to regions with an already successful vaccination campaign in Pakistan led to a stronger transmission of the poliovirus, again, in these regions. These migrations were not caused by the GPEI (hence not caused by the “problem solver”) but by political conflicts or military operations (Ahmad et al. 2015, and Basharat and Shaikh 2017) and thus can be considered as a dynamic factor.

Hence, constantly monitoring the situation with cognitive and metacognitive capacities was (and still is) strongly needed to remain on target along the journey to achieving the goal of eradicating polio globally. This adaptive problem-solving process involved, for example, searching for new information to figure out why these outbreaks kept flaring up despite the vaccination efforts and successes. These needed to be analyzed and evaluated to generate ideas and develop insightful, rational solutions. For instance, high population mobility and incomplete, outdated, and sometimes only hand-drawn maps of the affected regions were identified as decisive factors, as these maps led to the exclusion of entire settlements preventing a high vaccination rate in these regions (e.g., Barau et al. 2014). As a result of this insight, mapping and planning tools were applied and a robust global surveillance and response system was established. These coordinated immunization systems, including knowledge about the demographic structure of the population and its social, political, and religious background, have also served as a platform for other important health interventions and have been used to respond to other public health threats, such as COVID-19 and Ebola (Bill & Melinda Gates Foundation 2021).

Finally, the description of the problem space and the many social, political, and religious obstacles (Bill & Melinda Gates Foundation 2021) encountered during the problem-solving process should have already made it clear that ColPS skills were required at every step of the process. This seems intuitive, given the global collaboration among the different organizations and individuals involved in the GPEI, each with different areas of expertise and background knowledge. Similarly, communicating with local political and religious leaders as well as the volunteers requires the ability to engage in a process in which an attempt is made to solve a problem collaboratively.

## 5. Conclusions

The example of the GPEI clearly shows the many challenges in today’s world and the associated skills needed to address them. It also demonstrates the importance of problem-solving skills, as well as the importance of HOT skills associated with the problem-solving process (e.g., synthesizing, analyzing, reasoning, comprehending, applying, and evaluating information) for reaching a sustainable solution to consequential world problems. The success of the strategy, when fully implemented, was demonstrated by India’s success in stopping polio in January 2011, in one of the most technically difficult places to do so (WHO 2021). Through the efforts to eradicate polio globally, the GPEI paved the way to overcoming logistic, geographic, social, political, cultural, ethnic, gender, financial, and other barriers to working in the poorest and least accessible areas (Bill & Melinda Gates Foundation 2021). Moreover, the GPEI did more than advance the fight against polio. It created new ways to improve human health in developing countries through political commitment, funding, planning and management tools, and research (Bill & Melinda Gates Foundation 2021). In an extension of this process, which can also be described as a comprehensive problem-solving process, the knowledge and resources generated as part of the GPEI are now being transferred to address other global health threats, such as the COVID-19 pandemic. Hence, although the GPEI has not yet reached its final goal, it can serve as a successful example of why problem-solving and HOT skills are, in our opinion, one indispensable factor for such endeavors, although it goes without saying that these are not the only relevant skills or the universal solution to consequential world problems.

We want to stress, again, that HOT skills must be applied in a rational way when solving consequential world problems. That is, efficient ways of solving problems need to be considered and it needs to be evaluated whether solving strategies and beliefs are in line with the current evidence.

We would also like to emphasize an important implication that arises from the demonstrated relevance of HOT and problem-solving skills for addressing consequential world problems, namely the need to systematically integrate these skills into school curricula. We believe that schools need to go beyond preparing students to perform well on standardized tests, but rather prepare them to better meet the demands of a rapidly changing society characterized by accelerating globalization, the emergence of new digital technologies, and the rise of ever more challenging consequential world problems. Given the growing need for skills, such as collaboration, critical thinking, problem-solving, and the ability to acquire new skills and information (typically referred to as 21st-century skills; Ananiadou and Claro 2009 and the National Research Council 2012), it is not surprising that educational researchers and many contemporary educational curricula and assessment frameworks, such as the Programme for International Student Assessment (OECD 2004, 2013), call for coordinated efforts to integrate these skills into the curricula and better educate students in them. This is further underscored by the inclusion of problem-solving in PISA and the Programme for International Assessment of Adult Competencies (PIAAC; OECD 2012).

In conclusion, we believe that HOT and problem-solving skills form an essential cornerstone for addressing consequential world problems, and that systematically integrating these skills into school curricula will go a long way toward addressing global problems.

## Notes

1 Please note that this list should not be considered exhaustive.

2 The GPEI is a public-private partnership led by national governments with six partners: the World Health Organization; Rotary International; the US Centers for Disease Control and Prevention (CDC); the United Nations Children’s Fund (UNICEF); the Bill & Melinda Gates Foundation; and Gavi, the vaccine alliance.
